# Deep clinicopathological phenotyping identifies a previously unrecognized pathogenic *EMD* splice variant

**DOI:** 10.1002/acn3.51454

**Published:** 2021-09-15

**Authors:** Daniel G. Calame, Jawid M. Fatih, Isabella Herman, Zeynep Coban‐Akdemir, Haowei Du, Tadahiro Mitani, Shalini N. Jhangiani, Dana Marafi, Richard A. Gibbs, Jennifer E. Posey, Vidya P. Mehta, Carrie A. Mohila, Farida Abid, Timothy E. Lotze, Davut Pehlivan, Adekunle M. Adesina, James R. Lupski

**Affiliations:** ^1^ Division of Neurology and Developmental Neuroscience Department of Pediatrics Baylor College of Medicine Houston Texas 77030 USA; ^2^ Texas Children’s Hospital Houston Texas 77030 USA; ^3^ Department of Molecular and Human Genetics Baylor College of Medicine Houston Texas 77030 USA; ^4^ Human Genome Sequencing Center Baylor College of Medicine Houston Texas 77030 USA; ^5^ Department of Pediatrics Faculty of Medicine Kuwait University Safat 13110 Kuwait; ^6^ Department of Pathology Texas Children's Hospital Baylor College of Medicine Houston Texas 77030 USA; ^7^ Department of Pediatrics Baylor College of Medicine Houston Texas 77030 USA

## Abstract

Exome sequencing (ES) has revolutionized rare disease management, yet only ~25%–30% of patients receive a molecular diagnosis. A limiting factor is the quality of available phenotypic data. Here, we describe how deep clinicopathological phenotyping yielded a molecular diagnosis for a 19‐year‐old proband with muscular dystrophy and negative clinical ES. Deep phenotypic analysis identified two critical data points: (1) the absence of emerin protein in muscle biopsy and (2) clinical features consistent with Emery‐Dreifuss muscular dystrophy. Sequencing data analysis uncovered an ultra‐rare, intronic variant in *EMD*, the gene encoding emerin. The variant, NM_000117.3: c.188‐6A > G, is predicted to impact splicing by *in silico* tools. This case thus illustrates how better integration of clinicopathologic data into ES analysis can enhance diagnostic yield with implications for clinical practice.

## Introduction

Molecular diagnostic rates have significantly improved since the widespread implementation of exome sequencing (ES).^1^, ^2^ At present, the molecular diagnostic rate of ES obtained in clinical diagnostic laboratories is approximately 25%–30%, although higher or lower rates may be seen in certain disease states and patient populations.^3^, ^4^, ^5^, ^6^, ^7^, ^8^ Advances in ES data analyses including copy number variation (CNV) assessment,^9^, ^10^ better detection of insertion/deletion (indel) variant alleles,^11^ and homozygosity mapping using absence of heterozygosity (AOH) data as a surrogate measure of identity‐by‐descent (IBD)^10^ have improved diagnostic rates. Nevertheless, the notion of a “diagnostic ceiling” has been proposed because of similar diagnostic rates observed in multiple disease cohorts^5^, ^12^ as well as known limitations of ES technology (e.g., poor coverage of noncoding regions, limited detection of structural variants and repeat expansions).^13^

While both short read whole‐genome sequencing (SR‐WGS) and long‐read sequencing (LR‐WGS) technologies will likely improve molecular diagnostic rates by increasing coverage of noncoding regions, mounting evidence suggests the “molecular diagnostic gap” can be further narrowed by better integration of detailed phenotypic data into ES data analysis.^14^, ^15^, ^16^ Here, we provide an illustrative example of how a detailed analysis of extant clinicopathologic data led to a molecular diagnosis in a patient with muscular dystrophy and negative clinical ES (cES) and reflect on its heuristic implications for clinical practice.

## Methods

### Participants

All participants in this study provided informed consent as part of the Baylor‐Hopkins Center for Mendelian Genomics (BHCMG) initiative, including consent to publish photographs. This study was approved through Baylor College of Medicine Institutional Review Board (IRB) protocol H‐29697.

### Histology, immunofluorescence and western blot analysis of muscle samples

A vastus lateralis muscle biopsy was obtained during the proband’s clinical care. Hematoxylin and eosin (H&E) staining, immunostaining, and western blot analysis were performed by the Texas Children’s Hospital Neuropathology and Molecular Neuropathology Laboratory (Houston, TX) by board‐certified neuropathologists (CAM and AMA). For immunofluorescence, cryosections of skeletal muscle were stained using the nuclear stain 4′,6‐diamidino‐2‐phenylindole (DAPI) and antibodies against emerin or lamin A/C. Western blots were stained with antibodies for emerin and alpha‐sarcoglycan. Antibodies were obtained from Leica (emerin, cat. no. Emerin CE; alpha‐sarcoglycan, cat. no. A‐SARC‐L‐CE) or Abcam (lamin A/C, cat. no. AB5090).

### Exome sequencing

Research trio ES of genomic DNA obtained from peripheral blood was performed in the Baylor College of Medicine Human Genome Sequencing Center (BCM‐HGSC).^1^, ^17^ Rare variant family‐based exome analysis was performed as previously described.^1^, ^17^ Identified variants after computational parsing and filtering were experimentally confirmed and segregated via orthogonal Sanger dideoxy sequencing.

## Results

**Figure 1:**
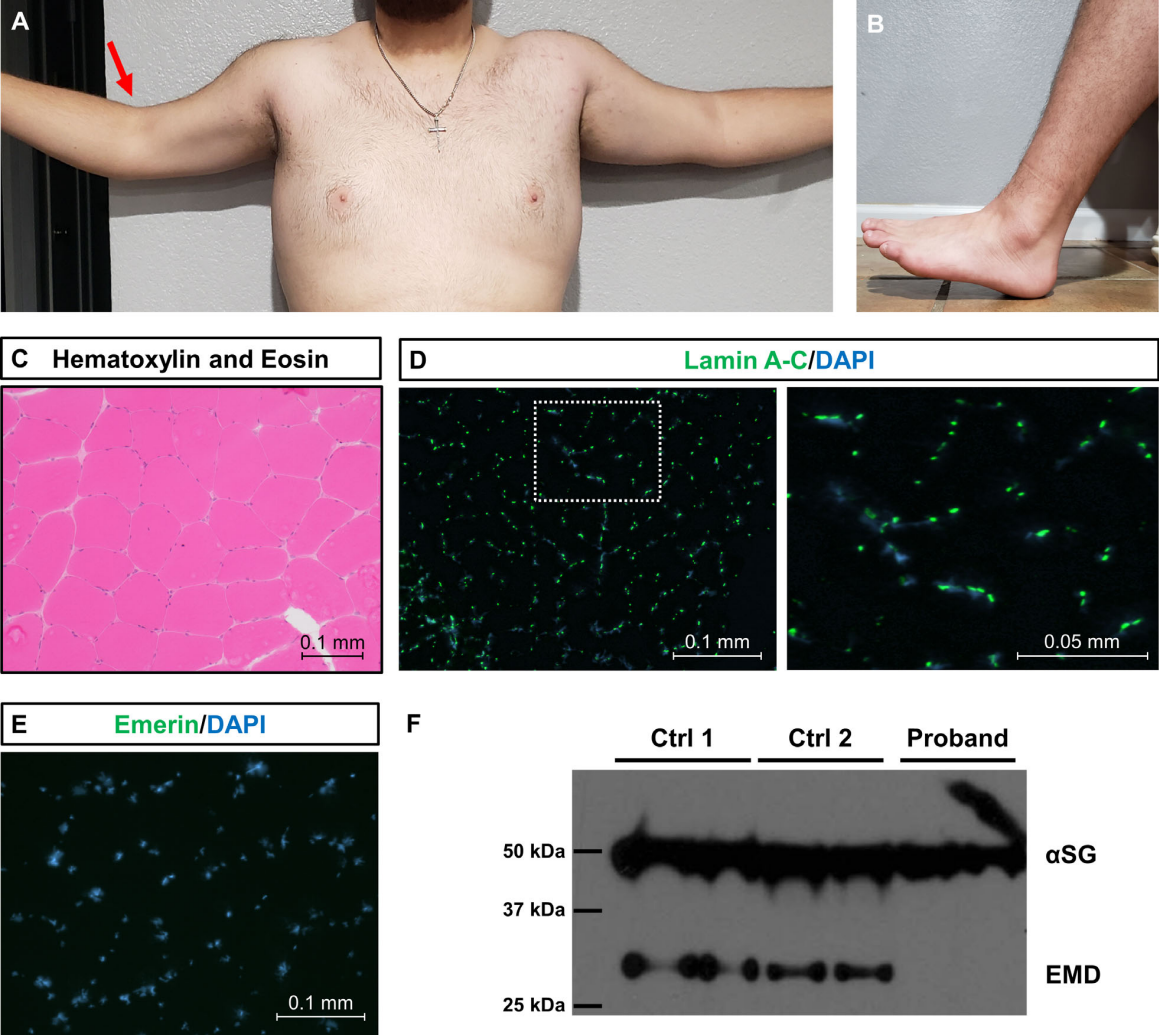
Clinicopathological features of proband with X‐linked Emery‐Dreifuss muscular dystrophy (A) Photograph of proband’s fully extended arms demonstrating limited active range of motion due to elbow contractures (right greater than left, red arrow). (B) Photograph of proband’s fully dorsiflexed foot demonstrating limited active range of motion due to ankle contractures. (C) Hematoxylin and eosin staining of left vastus lateralis muscle biopsy taken at 11 years old. (D) Immunofluorescence staining of lamin A‐C in the proband. Lamin A‐C is a nuclear protein involved in autosomal dominant Emery‐Dreifuss muscular dystrophy 1 (MIM #310300). Lamin A‐C (green) is widely expressed and co‐localizes with the nuclear stain DAPI (blue). (E) Immunofluorescence staining of emerin in the proband. Note the absence of emerin staining (green). Nuclei were counterstained with DAPI (blue). (F) Western blot of muscle biopsy protein lysates from proband and two control patients using antibodies for alpha‐sarcoglycan (αSG) and emerin (EMD). All samples were run in duplicate. Molecular weights are provided in kilodaltons (kDa). A 50 kDa band is seen in all samples corresponding to αSG. A 34 kDa band corresponding to EMD can be seen in the control samples but not in the proband.

The proband is a 19‐year‐old male with muscular dystrophy. Since early childhood he had frequent falls, easy fatigability, joint stiffness, and motor difficulties. Weakness and stiffness gradually progressed with age. On last physical examination at 18 years of age he had bilateral ankle contractures, elbow contractures (right greater than left), and weakness of bilateral ankle dorsiflexion, biceps, and hand interossei (Fig. 1A and B). There was no family history of neuromuscular disease. His creatine kinase (CK) levels trended upward with time (588 U/L at 17 years old, normal <245 U/L), suggesting a dystrophic condition. Needle electromyography showed small amplitude, short duration, polyphasic motor unit action potentials consistent with a myopathic condition. Muscle biopsy at age 11 years demonstrated unremarkable hematoxylin and eosin staining but absent emerin staining by immunofluorescence and western blot (Fig. 1C–F). Trio cES was subsequently performed (Baylor Genetics, BG, Laboratories, Houston, TX) and failed to identify any variants in *EMD* or other myopathy genes.

**Figure 2:**
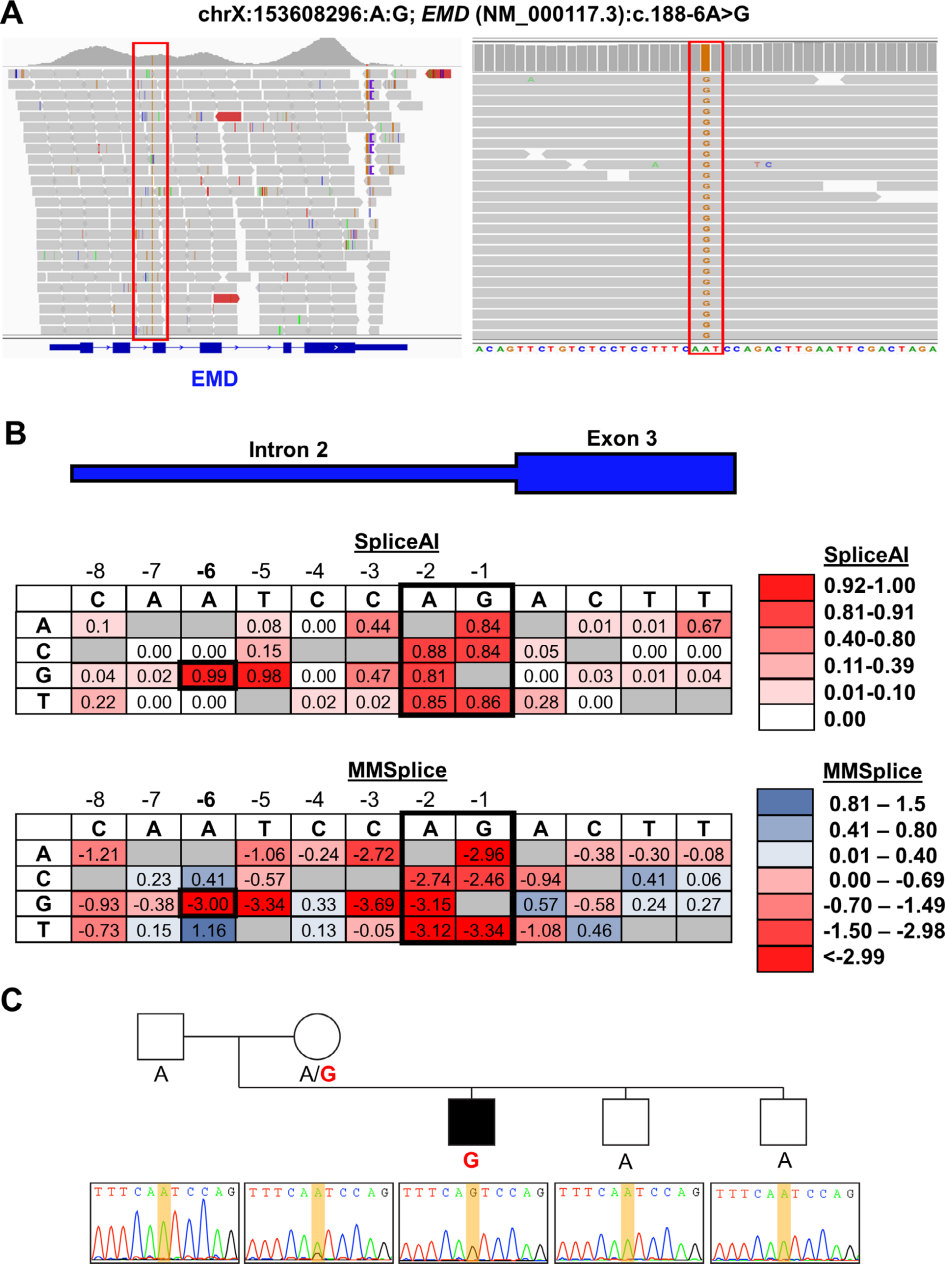
Research ES identifies a previously unrecognized *EMD* splice variant. (A) Screenshots of the proband’s BAM file data from Integrated Genomics Viewer (https://software.broadinstitute.org/software/igv/) showing a hemizygous intronic variant in intron 2 of *EMD*. (B) Heat map showing predicted impact on splicing from SpliceAI and MMSplice of all possible single‐nucleotide variants near c.188‐6A > G. Values in heat map were obtained from CADD v1.6 (https://cadd.gs.washington.edu/snv) and reflect the SpliceAI acceptor gain and MMSplice acceptor submodules, respectively. Darker shades of red indicate a greater predicted splicing impact. c.188‐6A > G and the consensus splice acceptor sites (−1, −2) are highlighted in black. (C) Sanger sequencing of the proband’s parents and unaffected brothers confirms the variant and demonstrates it is only found in the proband and his mother.

The proband and his family were subsequently enrolled in a “molecularly undiagnosed” neuromuscular disease cohort in the BHCMG and underwent research ES. Analysis began with an extensive review of the proband’s medical history, laboratory findings, electrodiagnostic studies, and muscle biopsy pathology. Due to negative emerin staining of muscle biopsy as well as phenotypic features consistent with Emery‐Dreifuss muscular dystrophy, the proband’s BAM file, a data file containing aligned sequencing data in a format which facilitates visualization, was inspected, leading to the identification of a hemizygous variant in *EMD* intron 2 (Fig. 2A). The variant, *EMD* (NM_000117.3):c.188‐6A > G, has a high CADD‐v1.6 score (20.6) and is predicted to impact splicing by multiple *in silico* algorithms (SpliceAI acceptor gain score 0.99; MMSp acceptor score −2.996; Human Splice Finder, alteration of the wild‐type acceptor site)^18^, ^19^, ^20^, ^21^ (Fig. 2B). The variant is absent from gnomAD v2.1.1.^22^ Segregation analysis within the family demonstrated that the variant was maternally inherited and absent from the proband’s unaffected brothers (Fig. 2C).

## Discussion

While diagnostic rates have improved since the advent of ES, many patients with presumed Mendelian disorders still lack a definitive molecular diagnosis. This diagnostic gap is often attributed to: (1) yet unidentified “disease‐contributing genes” and variant alleles and (2) the limited ability of ES to detect non‐coding and structural variants.^13^ By providing a complete sequence of all genic and intergenic regions, whole‐genome sequencing (WGS) has been regarded as a potential “panacea” and solution for the latter issue. However, WGS results to date have been underwhelming, with many additional diagnoses resulting from variants previously captured on ES and interim gene discoveries.^23^ Future hopes for resolving the diagnostic gap include LR‐WGS and RNA‐seq.^24^, ^25^, ^26^, ^27^ Although these approaches are promising, they will remain inaccessible in the clinic for the foreseeable future due to unavailability, high cost, and/or lack of appropriate tissue specimens.

An alternative hypothesis to explain the diagnostic gap is the under‐utilization of extant ES data. For example, expansion from proband‐only to trio ES improves diagnostic yield by permitting detection of *de novo* mutations and phasing (i.e., *cis* or *trans* configuration).^1^, ^28^ Copy number analysis of ES data, a practice not routinely performed by clinical diagnostic laboratories, may identify large deletions or duplications (>100 Kb) or even smaller homozygous exonic deletions.^9^, ^10^ The absence of heterozygosity (AOH) analysis, as a surrogate measure of runs‐of‐homozygosity (ROH), recognizes genomic intervals of identity‐by‐descent in families with or without a known history of consanguinity which prompts a thorough investigation of those regions for a potentially causative homozygous variant.^1^, ^28^, ^29^ Greater integration of clinicians and deep clinical phenotyping also improves diagnostic rates by enhancing variant prioritization and drawing increased scrutiny of extant single gene or gene families’ ES data.^14^, ^15^, ^16^ Deep phenotyping, the process of comprehensively assessing and categorizing individual phenotypic features often through Human Phenotype Ontology (HPO) terms, is routinely performed by medical geneticists and neurologists, yet the requisition forms for clinical genetic and genomic testing often fail to capture the depth of phenotyping performed by clinicians.^14^, ^30^ Finally, large amounts of “off‐target” sequencing data, for example intronic and 3′/5′ untranslated regions, are generated by ES yet are often filtered by cES bioinformatic pipelines despite increasing evidence of their significance in Mendelian disorders and improved *in silico* tools for evaluating their pathogenicity.^19^, ^21^, ^31^

Here we provide an illustrative example of how the incorporation of deep phenotyping into ES analysis improves molecular diagnostic yield. The proband carried a clinical diagnosis of muscular dystrophy with supporting laboratory and electrophysiologic data. The absence of emerin protein in his muscle biopsy strongly supported the clinical diagnosis of X‐linked Emery‐Dreifuss muscular dystrophy 1 (MIM #310300), and retrospective review of his clinical presentation identified compatible features including childhood‐onset joint contractures and slowly progressive muscle weakness. However, trio cES failed to identify pathogenic variants in *EMD* or other myopathy genes. Considering his clinical history and biopsy results, *EMD* sequencing data were reanalyzed, identifying a pathogenic hemizygous variant in *EMD*, c.188‐6A > G. The variant results in the substitution of a guanine for an adenine six nucleotides from the intron 2‐exon 3 boundary (Fig. 2B) which is predicted to create a new splice acceptor site by multiple *in silico* prediction tools. While the precise impact of the variant on splicing was not determined, the convergence of *in silico* algorithms predicting alteration of the splice acceptor site and the *in vivo* readout provided by western blot and immunofluorescence strongly suggests the mutant transcript either has a premature termination codon (PTC) resulting in nonsense‐mediated decay or encodes an unstable protein subject to rapid decay.

Identification of the variant had immediate clinical impact and management implications for the patient and his family. Gene therapy trials increasingly require a definitive genetic diagnosis for enrollment, and identification of a specific pathogenic intronic variant offers the opportunity for bespoke therapies like personalized antisense oligonucleotides (ASO). While personalized gene therapies may seem impractical, the recent story of milasen, an ASO customized and administered to a single patient with neuronal ceroid lipofuscinosis 7 (MIM # 610951), has demonstrated their feasibility and provide a pathway forward for rare disease.^32^ Additionally, these studies identified the carrier status of the proband’s mother, a finding of considerable significance as female carriers can develop cardiac conduction defects and are at risk of sudden death.^33^ Therefore, cardiology follow‐up and screening of the extended family was recommended.

The importance of intronic variants such as *EMD* c.188‐6A > G which impact *cis*‐acting elements in human disease is well‐recognized.^34^, ^35^, ^36^ The proportion of human pathogenic variants disrupting *cis*‐acting elements has been estimated between 15% to 60%.^34^, ^35^ Prior to this report, only a single non‐consensus splice variant, *EMD*: c.449 + 23_450−35del, was recognized.^37^ Located within intron 5, the variant was detected on a neuromuscular gene panel and would have been well‐covered in the BCM‐HGSC ES platform (Fig. 2A). Studies of *EMD* constructs with variably sized intron 5 deletions demonstrated the 23‐nucleotide deletion does not impact the major branchpoint c.450‐24A but rather causes splicing abnormalities due to excessive intronic shortening.^37^ Such an intron size constraint mutational mechanism may disproportionately affect genes with small size introns and remains underappreciated despite the fact that it was described over a decade ago.^38^ Additional pathogenic intronic *EMD* variants will undoubtedly be identified with increased implementation of WGS and closer scrutiny of extant ES data. Further identification and study of pathogenic intronic variants through ES/GS, mini‐gene assays, and RNA‐seq will clarify the mechanisms involved in splicing and in turn improve *in silico* predictive models.

In summary, this report illustrates how the integration of deep clinicopathological phenotypic data into ES analysis improves molecular diagnostic yield. Clinicians play a critical role in this process by providing accurate and detailed clinical data to clinical diagnostic laboratories and following up on all exome negative studies. Additionally, an active dialogue between clinicians and laboratories is essential to maximize diagnostic yield.

## Conflict of Interest

J.R.L. has stock ownership in 23andMe, is a paid consultant for Regeneron Genetics Center, and is a co‐inventor on multiple United States and European patents related to molecular diagnostics for inherited neuropathies, eye diseases, and bacterial genomic fingerprinting. The Department of Molecular and Human Genetics at Baylor College of Medicine receives revenue from clinical genetic testing conducted at Baylor Genetics (BG) Laboratories; JRL is a member of the Scientific Advisory Board of BG. Other authors have no potential conflicts to report.

## Data Sharing

All data supporting the findings of this study are available from authors DGC and JRL upon reasonable request.
